# A NATIONAL ASSESSMENT OF THE RELATIONSHIP BETWEEN NURSING HOME CULTURE CHANGE AND RESIDENT OUTCOMES

**DOI:** 10.1093/geroni/igz038.239

**Published:** 2019-11-08

**Authors:** Margot L Schwartz, Julie C Lima, Pedro L Gozalo, Melissa A Clark, Susan C Miller

**Affiliations:** 1 Brown University School of Public Health, Providence, Rhode Island, United States; 2 Brown University, Providence, Rhode Island, United States

## Abstract

Literature is mixed regarding the relationship between Nursing Home (NH) culture change and resident outcomes, and the majority of studies are limited to small samples. We evaluated this relationship separately for five unique domains of NH culture change (physical environment, resident care, staff empowerment, leadership, and family and community involvement practices) using a 2016/2017 survey administered to a stratified-random national sample of NHs; 74% of NH administrators responded (n=1,583). We assessed the relationship between each culture change domain and 8 outcomes (calculated with MDS 3.0 and Medicare claims data) using resident-level multivariable logistic regression models, that accounted for resident and NH characteristics, and were weighted by facility-level inverse probability weights (to address NH Selection). We found the relationship between NH culture change and resident outcomes varied by culture change domain. High scores on leadership practices (i.e., two-way communication, staff involvement, education/training, respect for workers, and coaching) were most strongly associated with outcomes. Compared to the lowest quartile, performance in the highest quartile (most implementation of practices) on the leadership domain was associated with 13% lower odds (OR: 0.87, 95%CI: 0.78, 0.96) of urinary tract infections, 15% lower odds (OR: 0.85, 95%CI: 0.80, 0.91) of worsened locomotion, and 41% lower odds (OR: 0.59, 95%CI: 0.42, 0.83) of physical restraint use. For the other domains the estimates (and statistical significance) of the relationship with outcomes varied more than observed for leadership. Our findings emphasize the importance of high-quality NH leadership. Investments in improved leadership practices may result in higher-quality resident outcomes.

